# A Transgenic *Camelina sativa* Seed Oil Effectively Replaces Fish Oil as a Dietary Source of Eicosapentaenoic Acid in Mice^1^,^2^,^3^

**DOI:** 10.3945/jn.115.223941

**Published:** 2016-01-20

**Authors:** Noemi Tejera, David Vauzour, Monica B Betancor, Olga Sayanova, Sarah Usher, Marianne Cochard, Neil Rigby, Noemi Ruiz-Lopez, David Menoyo, Douglas R Tocher, Johnathan A Napier, Anne Marie Minihane

**Affiliations:** 4 Department of Nutrition, Norwich Medical School, Faculty of Medicine and Health Sciences, University of East Anglia, Norwich, United Kingdom;; 5 Institute of Food Research, Norwich Research Park, Norwich, United Kingdom;; 6 Institute of Aquaculture, School of Natural Sciences, University of Stirling, Stirling, United Kingdom;; 7 Department of Biological Chemistry and Crop Protection, Rothamsted Research, Harpenden, United Kingdom; and; 8 Department of Agricultural Production, School of Agricultural Engineering, Technical University of Madrid, Madrid, Spain

**Keywords:** n–3 PUFA, EPA, DHA, *Camelina* oil, fish oil, sustainability, desaturation, *Fads*, transgenic, TG sn-2

## Abstract

**Background:** Fish currently supplies only 40% of the eicosapentaenoic acid (EPA) and docosahexaenoic acid (DHA) required to allow all individuals globally to meet the minimum intake recommendation of 500 mg/d. Therefore, alternative sustainable sources are needed.

**Objective:** The main objective was to investigate the ability of genetically engineered *Camelina sativa* (20% EPA) oil (CO) to enrich tissue EPA and DHA relative to an EPA-rich fish oil (FO) in mammals.

**Methods:** Six-week-old male C57BL/6J mice were fed for 10 wk either a palm oil–containing control (C) diet or diets supplemented with EPA-CO or FO, with the C, low-EPA CO (COL), high-EPA CO (COH), low-EPA FO (FOL), and high-EPA FO (FOH) diets providing 0, 0.4, 3.4, 0.3, and 2.9 g EPA/kg diet, respectively. Liver, muscle, and brain were collected for fatty acid analysis, and blood glucose and serum lipids were quantified. The expression of selected hepatic genes involved in EPA and DHA biosynthesis and in modulating their cellular impact was determined.

**Results:** The oils were well tolerated, with significantly greater weight gain in the COH and FOH groups relative to the C group (*P* < 0.001). Significantly lower (36–38%) blood glucose concentrations were evident in the FOH and COH mice relative to C mice (*P* < 0.01). Hepatic EPA concentrations were higher in all EPA groups relative to the C group (*P* < 0.001), with concentrations of 0.0, 0.4, 2.9, 0.2, and 3.6 g/100 g liver total lipids in the C, COL, COH, FOL, and FOH groups, respectively. Comparable dose-independent enrichments of liver DHA were observed in mice fed CO and FO diets (*P* < 0.001). Relative to the C group, lower fatty acid desaturase 1 (*Fads1*) expression (*P* < 0.005) was observed in the COH and FOH groups. Higher fatty acid desaturase 2 (*Fads2*), peroxisome proliferator–activated receptor α (*Ppara*), and peroxisome proliferator–activated receptor γ (*Pparg*) (*P* < 0.005) expressions were induced by CO. No impact of treatment on liver X receptor α (*Lxra*) or sterol regulatory element-binding protein 1c (*Srebp1c*) was evident.

**Conclusions:** Oil from transgenic *Camelina* is a bioavailable source of EPA in mice. These data provide support for the future assessment of this oil in a human feeding trial.

## Introduction

Although randomized controlled trials have not been completely consistent, a large body of human prospective cohort evidence, as well as animal feeding studies, have shown the beneficial impact of the n–3 (ω-3) long-chain (LC)^9^ PUFAs EPA (20:5n–3) and DHA (22:6n–3) on fetal development and cardiovascular and cognitive health (1–4). In humans, EPA, and to a lesser extent DHA, can be synthesized de novo from their precursor essential FA, α-linolenic acid (ALA; 18:3n–3), which is particularly rich in several seed oils. However, this bioconversion, in which elongation of very long chain fatty acids proteins (*ELOVL*s; encoded by *ELOVL2* and *ELOVL5*) and Δ5 and Δ6 desaturases [encoded by FA desaturases (*FADS*) 1 (*FADS1*) and 2 (*FADS2*)] are key metabolizing enzymes (5–7), is inefficient (8, 9). Therefore, based largely on their cardiovascular benefits, international and national health organizations and societies such as the International Society for the Study of Fatty Acids and Lipids (10), the UK Scientific Advisory Committee in Nutrition (11), and the American Heart Association (12) recommend a minimum dietary intake of 500 mg preformed EPA+DHA/d (achieved through the consumption of 1 to 2 portions of oily fish per week), increasing to 1 g or 2–4 g/d for the secondary prevention of cardiovascular diseases or as a TG-lowering therapy, respectively.

It has been estimated that, globally, ∼1.3 million metric tons of EPA plus DHA per annum are needed to allow all individuals to have a minimum intake of 500 mg/d. Current sources, which are almost exclusively derived from fish, provide only 40% of this (13) [of which >75% is used in aquaculture (14)], leaving a large deficit between supply and need. Zooplankton and microalgae have been investigated (15); however, the availability of EPA and DHA via these sources presents substantial technological and cost challenges, which are generally limiting their use. Alternatively, seed oils rich in stearidonic acid (SDA; 18:4n–3), an intermediate in the synthesis of EPA from ALA, have also been considered as a possible alternative to fish oil (FO). Supplementation with a genetically engineered soybean oil to contain SDA resulted in increased EPA, but not DHA, concentrations in human plasma and erythrocytes (16, 17). In aquaculture, the partial replacement of FO with either SDA-soybean oil or *Echium *oil (naturally rich in SDA) was shown to have ambiguous effects on growth and performance and did not enhance tissue n–3 LC-PUFA concentrations, although higher tissue SDA was evident (which may ultimately improve human EPA status) (18–21). Therefore, overall, SDA-rich oils have not emerged as an adequate replacement for FO.

Due to the scalability of agriculture-based sources, metabolic engineering of oilseed crops (which are naturally devoid of EPA and DHA) to accumulate these FAs is emerging as one of the most practical solutions to n–3 LC-PUFA supply (22–24). *Camelina sativa* is a Brassicaceae crop that naturally has a high content (45%) of the precursor ALA. By using transgenic technology and the introduction of heterologous genes (from microalgae) of the biosynthetic pathway and their regulators into *Camelina*, this oilseed has been genetically engineered to produce oils containing up to 30% EPA or EPA+DHA (25–27). One of these transgenic seed oils, containing 20% of total FAs as EPA, was recently used as a replacement for FO in the feeds of Atlantic salmon (*Salmo salar*), showing comparable tissue EPA enrichment to FO (28). The impact on EPA and DHA status upon direct feeding to humans is currently unknown for this oil.

The aim of the present study was to compare, for the first time to our knowledge, how equal doses of EPA provided as EPA-enriched *Camelina* oil (CO) or EPA-rich concentrated FO affect tissue FA status in mammals (C57BL/6J mice). Because the position at which PUFAs are complexed on the glycerol backbone of TGs (*sn*1,3) has been shown to affect their bioavailability and bioefficacy (29, 30), regiospecific analysis of the CO and FO was performed. Gene expressions of the *Elovl* and *Fads* genes along with transcription factors that modulate the cellular effects of EPA and DHA, namely peroxisome proliferator–activated receptor (*Ppar*) α (*Ppara*) and γ (*Pparg*), sterol regulatory element-binding protein 1c (*Srebp1c*), and liver X receptor α (*Lxra*) (31), were also investigated.

## Methods

### 

#### Animals and design.

Experimental procedures and protocols used in this study were reviewed and approved by the Animal Welfare and Ethical Review Body and were conducted within the provisions of the Animals (Scientific Procedures) Act of 1986 (32), under project license number 80/2533 and following the Laboratory Animal Science Association Guiding Principles for Preparing for and Undertaking Aseptic Surgery (33).

Male C57BL/6J mice (*n* = 40; 25.9 ± 0.4 g), aged 6 wk, were purchased from Charles River Laboratories (Margate, United Kingdom). Mice were housed 4 per cage and under controlled temperature (21° ± 2°C) and humidity (55% ± 10%) and a standard light-dark cycle (12 h/12 h) and consumed food and water ad libitum throughout. After a 2-wk acclimatization period of consuming a standard RM3 diet (Rat and Mouse No. 3 Breeding Diet; Special Diets Services) (34), which provided 11% of energy from fat, 27% from protein, and 62% from carbohydrates, mice (*n* = 8/group) were allocated to 1 of the 5 following feed pellet test diets for 10 wk: control (C), EPA-rich *Camelina* oil, low dose (COL), EPA-rich *Camelina* oil, high dose (COH), EPA-rich Fish oil, low dose (FOL), or EPA-rich Fish oil, high dose (FOH). The nutrient and FA compositions of the experimental diets are given in **Supplemental Tables 1–3**.

Mice were weighed once a week, and food intake was recorded 3 times/wk. The diets were frozen in batches at −20°C and defrosted and replaced every other day to ensure minimal oxidation of the FAs. At the end of the 10-wk intervention, food was removed for 16 h. The mice were anesthetized by using 4% isoflurane in medical oxygen, and blood was drawn by cardiac puncture into serum separation tubes (Becton Dickinson). Glucose concentrations were measured in whole blood by using an AlphaTRAK 2 glucose meter (Abbott Laboratories Ltd.). Blood samples were then allowed to clot for 30 min at room temperature before serum was separated by centrifugation (10 min, 1300 × *g*, at room temperature). Mice were transcardially perfused with an ice-cold saline solution containing sodium heparin (10 U/mL). Livers were removed, rinsed with ice-cold 150 mmol NaCl/L, blotted, and weighed. The right lobes were stored in RNALater (Sigma-Aldrich) for gene expression analysis. The remainder of the liver, muscle (gastrocnemius), and brain samples were snap-frozen, and all samples were stored at −80°C for further analysis. These tissues were chosen due to their central role in EPA and DHA biosynthesis (liver), FA utilization (muscle), and high n–3 PUFA, and in particular DHA, content (brain).

#### Mouse diets.

All diets were based on the TestDiet AIN-93M semipurified diet 58M1 for rodents (TestDiet Europe; IPS Product Supplies Ltd.) and provided 10% of energy from fat, 20% from protein, and 70% from carbohydrates (Supplemental Tables 1 and 2). The control diet contained palm oil (PO; William Hodgson & Co.), which was chosen as a high-saturated-fat oil source, typical of a Western-type human diet. Furthermore, PO is low in ALA, thereby limiting endogenous EPA synthesis in the mouse tissue. Details of the metabolic engineering, production, and composition of the transgenic EPA-rich CO have been published previously (35, 36), with the oil providing 20% EPA, 0% DHA, and 16%, 17%, and 66% of total SFAs, MUFAs, and PUFAs, respectively.

The COL and COH diets contained 0.31% and 2.15% of transgenic EPA-rich CO and the FOL and FOH diets contained 0.08% and 0.57% of an EPA-rich FO (EPAX 6015 TG, EPA:DHA-4:1; Epax A/S). The oil content of all 4 diets was taken up to 10% of energy with the use of PO. This resulted in C, COL, COH, FOL, and FOH diets containing 0, 0.4, 3.4, 0.3, and 2.9 g EPA/kg diet, respectively.

These amounts of oils were chosen to provide human equivalent doses (HEDs) of EPA of 500 mg COL and FO/d and 3.5 g COH and FOH/d, which represent the minimal recommended EPA+DHA intake (500 mg/d) and the 2- to 4-g/d dose, respectively, recommended by the American Heart Association for TG lowering (12). The equivalent animal doses (AD) were determined on the basis of allometric scaling and body surface area–based calculations considering the HED in a normal-weight adult (70 kg) with the use of the following formula: HED (mg/kg) = AD (mg/kg) × [weight mouse (kg)/weight human (kg)]^(0.33)^ and assuming a daily food intake of 5 g/d (37).

#### Lipid blood tests and FA analysis.

Serum concentrations of total cholesterol (TC), HDL cholesterol, and TGs in feed-deprived mice were quantified with commercially available kits from Instrumentation Laboratories on a clinical chemistry analyzer IL650 (Instrumentation Laboratories). Total lipids (TLs) were extracted from 200 mg ground diets, 150 mg liver, 200 mg brain, and 100 mg muscle samples by using chloroform/methanol (2:1 vol:vol) containing 0.01% BHT as an antioxidant. FAs were analyzed by GC-Flame Ionization Detector following the method described previously (38).

#### RNA isolation and real-time qPCR.

Total RNA was isolated from the livers of 6 mice per dietary treatment after being homogenized in 1 mL TriReagent (Sigma-Aldrich), and cDNA was synthesized as detailed in Betancor et al. (39). The expression of genes of interest (**Supplemental Table 4**) was determined by qPCR, normalizing the results against β-actin. qPCR was performed by using a Biometra TOptical Thermocycler (Analytik Jena).

#### TG purification and positional analysis.

The TG fraction from the experimental oils was separated by thin layer chromatography as previously described (40). FAME composition of these TG fractions was obtained by heating the samples in methanol:toluene:H_2_SO_4_ (88:10:2, by volume) (41). Positional analysis of purified TGs was performed according to Ruiz-Lopez et al. (26).

#### Statistical analysis.

Body weight, food intake, blood glucose, serum lipids, and tissue FAs are presented as means ± SEMs. The data were checked for normality of distribution by using the Kolmogorov-Smirnoff test and log-transformed when necessary. Homogeneity of variance was established by using the Levene test. Statistical analyses of body weight and food intake were performed by using 2-factor ANOVA with repeated measures followed by Bonferroni’s multiple-comparison tests. One-factor ANOVA was used to test for the effects of diet on serum lipids and tissue FA compositions, measured at 10 wk. Tukey’s multiple-comparison tests were used to establish intergroup differences when the *F* value was significant. The statistical analysis was performed by using the SPSS package (version 16.0; SPSS, Inc.), with *P* < 0.05 taken as being significant. Gene expression results were analyzed by using the relative expression software tool (REST) (42), which uses a pairwise fixed reallocation randomization test (10,000 randomizations) with efficiency correction.

## Results

### 

#### Body weight and food intake.

**FIGURE 1:**
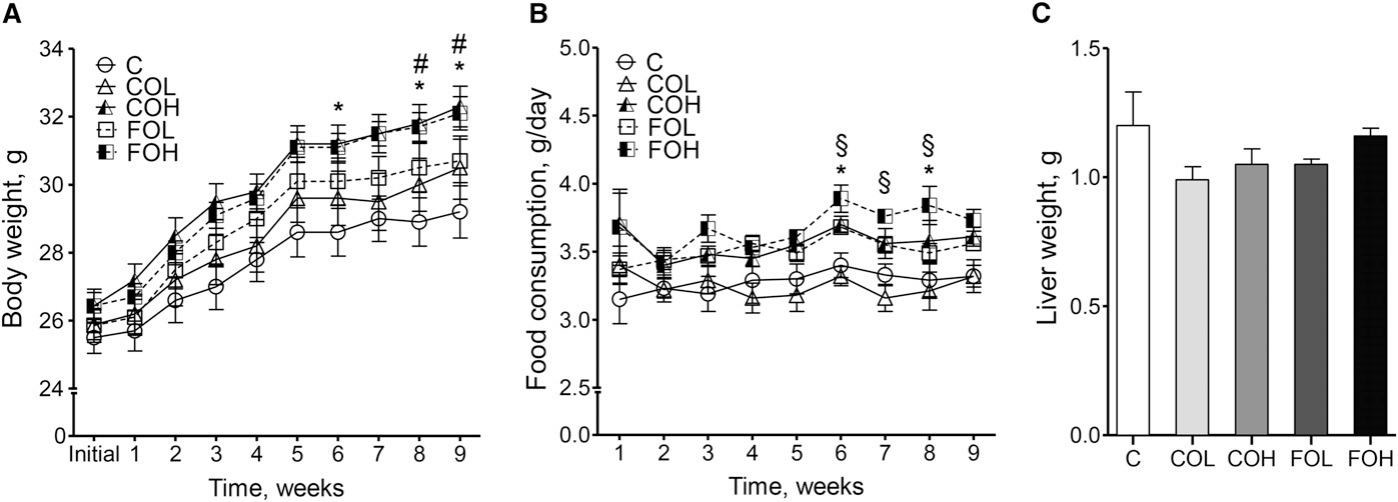
Body weight (A), food consumption (B), and liver weight (C) in male C57BL/6J mice after 10 wk of feeding diets providing EPA as EPA-rich transgenic *Camelina* oil or EPA-rich fish oil relative to a control diet. Values are means ± SEMs, *n* = 8. *^,#,§^Differences between groups (*P* < 0.05, 2-factor ANOVA with repeated measures followed by Bonferroni’s multiple-comparison tests): *C compared with FOH, ^#^C compared with COH, ^§^COL compared with FOH. Week 10 data are not presented in panels A and B because mice had their food removed for 16 h before killing, and therefore the data are not directly comparable to weeks 1–9. C, control diet; COH, EPA-rich *Camelina* oil, high dose; COL, EPA-rich *Camelina* oil, low dose; FOH, EPA-rich fish oil, high dose; FOL, EPA-rich fish oil, low dose.

There were no mouse deaths or indications of ill health over the 10-wk intervention period, with no impact of treatment on liver weight (**Figure 1C**). All of the groups had a significant increase in body weight, ranging from 14% for the C group to 21–22% for the FOH and COH groups (*P* = 0.002), with the change from baseline being significantly higher in the COH and FOH groups relative to the C group (Figure 1A). A significant difference in body weight (*P* < 0.005) was observed between C and FOH groups from 6 wk onward, which was associated with higher food consumption in the FOH group compared with the C group (*P* < 0.001) (Figure 1B). A higher body weight was also evident in the COH mice than in C mice (*P* < 0.005) from week 8 onward, which was not reflected in differences in food intake.

#### Diet analysis.

Both EPA-rich CO diets (COL and COH) had a higher content of linoleic acid (LA; 18:2n–6), total n–6 PUFAs, and ALA than did the C or FOL and FOH diets (Supplemental Table 3). These results are reflective of the presence of *Camelina sativa,* a naturally C18-PUFA–rich oil.

The amounts of EPA were comparable in both the COL and FOL (0.9% and 0.6%) and the COH and FOH (8.3% and 7.1%) diets, with 0.1% and 2.3% DHA present in the FOL and FOH diets as a result of the inclusion of FO. The regiospecific analysis of the TG fraction in the experimental oils showed that almost all of the palmitic acid (16:0) and stearic acid (18:0) were in the *sn*-2 position in the FO, compared with ∼20% in the CO. Comparable proportions of EPA were present in *sn*-2 in the FO (46.0%) and CO (51.5%) (**Supplemental Table 5**).

#### Fasting glucose and blood lipids.

**FIGURE 2:**
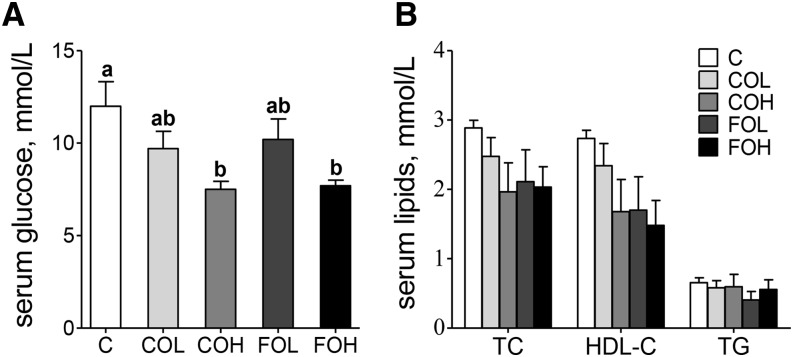
Effects of 10 wk of feeding diets providing EPA as EPA-rich transgenic *Camelina* oil or EPA-rich fish oil relative to a control diet on serum glucose (A) and lipids (B) in male C57BL/6J mice. Food was removed for 16 h before blood sample collection. Values are means ± SEMs, *n* = 8. Labeled means without a common letter differ, *P* < 0.05 (1-factor ANOVA followed by Tukey’s multiple-comparison tests). C, control diet; COH, EPA-rich *Camelina* oil, high dose; COL, EPA-rich *Camelina* oil, low dose; FOH, EPA-rich fish oil, high dose; FOL, EPA-rich fish oil, low dose; HDL-C, HDL cholesterol; TC, total cholesterol.

Fasting blood glucose concentrations were lower in the COH- and FOH-fed mice relative to C mice (*P* = 0.01) (**Figure 2A**). No significant impact of treatment on fasting serum TC, TGs, or HDL cholesterol was observed (Figure 2B).

#### FA composition.

The PUFA composition of the TLs in the liver (**Table 1**) reflected, in general terms, the composition of the diets. Significantly higher concentrations of EPA were evident in all of the experimental groups relative to the C group (*P* < 0.001), with EPA enrichments in the following order: FOH = COH > COL > FOL > C. DHA biosynthesis was evident in all of the groups, with the concentrations of this FA 3–4 times higher in mice fed the experimental diets compared with the C group (*P* < 0.001). The liver TL total n–3 PUFA concentrations were in the following order: COH = FOH > COL = FOL > C (*P* < 0.001). The concentrations of LA were higher in all of the experimental groups compared with the C group (*P* < 0.000). Mice fed the C diet showed higher concentrations of metabolites derived from LA (20:2n–6, 20:3n–6, and 20:4n–6; 1.2%, 0.7%, and 8.2%, respectively) when compared with FOH mice (0.1%, 0.3%, and 2.8%, respectively). SFA percentages were similar between groups, with the exception of palmitic acid (*P* < 0.001).

**TABLE 1:** Main FAs (≥0.5 g/100 g FAs) in liver total lipids of male C57BL/6J mice after 10 wk of feeding diets providing EPA as EPA-rich transgenic *Camelina* oil or EPA-rich fish oil relative to a control diet^1^

	Diet, g/100 g FAs					
FAs	C	COL	COH	FOL	FOH	*P*^2^
14:0	0.5 ± 0.0	0.5 ± 0.1	0.6 ± 0.0	0.5 ± 0.1	0.6 ± 0.1	NS
16:0	22.2 ± 0.3^d^	23.6 ± 0.5^c,d^	25.7 ± 0.4^a,b^	24.3 ± 0.3^b,c^	26.4 ± 0.7^a^	<0.001
16:1n–7	6.4 ± 0.4	6.2 ± 0.5	5.1 ± 0.4	5.5 ± 0.6	6.0 ± 0.5	NS
18:0	5.9 ± 0.4	5.5 ± 0.5	6.3 ± 0.5	7.2 ± 0.7	5.7 ± 0.4	NS
18:1^3^	43.9 ± 1.4^a^	36.9 ± 1.1^b^	26.6 ± 0.8^c^	35.4 ± 1.5^b^	33.2 ± 0.7^b^	<0.001
18:2n–6	6.6 ± 0.4^d^	9.2 ± 0.4^b,c^	13.4 ± 0.4^a^	7.8 ± 0.3^c,d^	9.4 ± 0.4^b^	<0.001
18:3n–3	0.0 ± 0.0^c^	0.2 ± 0.0^b^	1.2 ± 0.1^a^	0.0 ± 0.0^c^	0.1 ± 0.0^b^	<0.001
20:1n–9	0.7 ± 0.1^a^	0.2 ± 0.1^b^	0.4 ± 0.1^a,b^	0.2 ± 0.1^b^	0.1 ± 0.0^b^	<0.001
20:2n–6	1.2 ± 0.1^a^	0.6 ± 0.0^c^	0.3 ± 0.0^d^	0.8 ± 0.1^b^	0.1 ± 0.0^e^	<0.001
20:3n–6	0.7 ± 0.1^a^	0.7 ± 0.1^a^	0.5 ± 0.0^a^	0.8 ± 0.1^a^	0.3 ± 0.0^b^	<0.001
20:4n–6	8.2 ± 0.7^a^	7.5 ± 0.0^a^	6.4 ± 0.5^a^	8.8 ± 0.8^a^	2.8 ± 0.2^b^	<0.001
20:5n–3	0.0 ± 0.0^d^	0.4 ± 0.0^b^	2.9 ± 0.1^a^	0.2 ± 0.0^c^	3.6 ± 0.2^a^	<0.001
24:1	0.1 ± 0.0^c^	0.4 ± 0.0^b^	1.6 ± 0.3^a^	0.3 ± 0.0^b^	1.3 ± 0.3^a,b^	0.001
22:6n–3	2.4 ± 0.1^b^	7.2 ± 0.8^a^	7.7 ± 0.6^a^	7.2 ± 0.6^a^	9.5 ± 0.5^a^	<0.001
Total n–3 PUFAs	2.8 ± 0.2^c^	8.0 ± 0.8^b^	12.2 ± 0.7^a^	7.7 ± 0.6^b^	13.4 ± 0.7^a^	<0.001
Total n–6 PUFAs	17.0 ± 1.0^b^	18.3 ± 0.9^a,b^	20.9 ± 0.6^a^	18.5 ± 0.9^a,b^	12.7 ± 0.5^c^	<0.001
n–3 to n–6 PUFA ratio	0.2 ± 0.0^d^	0.4 ± 0.0^c^	0.6 ± 0.0^b^	0.4 ± 0.0^c^	1.0 ± 0.0^a^	<0.001

1 Values are means ± SEMs, *n* = 8. Totals include some minor components not shown. Labeled means in a row without a common letter differ, *P* < 0.05. C, control; COH, EPA-rich *Camelina* oil, high dose; COL, EPA-rich *Camelina* oil, low dose; FOH, EPA-rich fish oil, high dose; FOL, EPA-rich fish oil, low dose.

2 Derived by using 1-factor ANOVA followed by Tukey’s multiple-comparison tests.

3 Contains n–9 and n–7 isomers.

In brain tissue (**Table 2**), the expected low concentrations of EPA (0.2%) were observed, with no impact of diet. Relative to all other groups, modestly higher DHA concentrations were evident after the FOH intervention (*P* = 0.006), with associated lower docosapentaenoic acid (22:5n–3) (except when compared with FOL; *P* < 0.019) and arachidonic acid (AA; 20:4n–6; *P* < 0.001). A significant impact of intervention on total n–3 PUFAs (*P* = 0.004) and n–3 to n–6 PUFA ratio (*P* < 0.001) was also evident. No significant impact of treatment on the SFA content of brain tissue was observed.

**TABLE 2:** Main FAs (≥0.5 g/100 g FAs) in brain total lipids of male C57BL/6J mice after 10 wk of feeding diets providing EPA as EPA-rich transgenic *Camelina* oil or EPA-rich fish oil relative to a control diet^1^

	Diet, g/100 g FAs					
FAs	C	COL	COH	FOL	FOH	*P*^2^
16:0	20.2 ± 0.1	20.0 ± 0.4	19.9 ± 0.3	20.6 ± 0.2	20.8 ± 0.1	NS
16:1n–7	0.6 ± 0.0	0.7 ± 0.0	0.7 ± 0.0	0.7 ± 0.0	0.7 ± 0.0	NS
18:0	20.8 ± 0.2	20.1 ± 0.6	20.6 ± 0.2	20.8 ± 0.2	21.0 ± 0.2	NS
18:1^3^	24.1 ± 0.5	23.8 ± 0.5	23.9 ± 0.3	24.5 ± 0.3	24.5 ± 0.6	NS
18:2n–6	0.3 ± 0.0^b^	0.5 ± 0.0^a^	0.5 ± 0.0^a^	0.5 ± 0.0^a^	0.3 ± 0.1^a,b^	0.043
18:4n–3	0.7 ± 0.1	0.4 ± 0.0	0.5 ± 0.0	0.9 ± 0.2	0.8 ± 0.2	NS
20:1n–9	2.6 ± 0.2^a^	1.9 ± 0.3^a^	1.2 ± 0.4^a,b^	0.5 ± 0.0^b^	0.5 ± 0.0^b^	0.002
20:3n–6	0.4 ± 0.0^b^	0.5 ± 0.0^a^	0.5 ± 0.0^a^	0.5 ± 0.0^a^	0.5 ± 0.0^a^	<0.001
20:4n–6	9.7 ± 0.1^a^	9.6 ± 0.1^a^	8.9 ± 0.2^a^	9.3 ± 0.1^a^	7.2 ± 0.2^b^	<0.001
20:4n–3	0.3 ± 0.0	0.4 ± 0.0	0.4 ± 0.0	0.4 ± 0.0	0.4 ± 0.0	NS
20:5n–3	0.2 ± 0.0	0.2 ± 0.0	0.2 ± 0.0	0.2 ± 0.0	0.2 ± 0.0	NS
22:5n–3	2.2 ± 0.1^a^	2.2 ± 0.0^a^	2.2 ± 0.0^a^	2.2 ± 0.2^a,b^	1.8 ± 0.0^b^	0.019
24:1	0.1 ± 0.0^c^	0.3 ± 0.0^b^	0.7 ± 0.0^a^	0.1 ± 0.0^b,c^	0.8 ± 0.0^a^	<0.001
22:6n–3	15.2 ± 0.3^b^	16.8 ± 0.6^a,b^	16.4 ± 0.2^a,b^	16.4 ± 0.2^a,b^	17.7 ± 0.2^a^	0.006
Total n–3 PUFAs	18.7 ± 0.2^b^	20.0 ± 0.6^a,b^	19.7 ± 0.2^a,b^	20.1 ± 0.3^a,b^	20.8 ± 0.2^a^	0.004
Total n–6 PUFAs	11.0 ± 0.1^a^	11.0 ± 0.2^a^	10.1 ± 0.2^b^	10.8 ± 0.1^a,b^	8.4 ± 0.2^c^	<0.001
n–3 to n–6 PUFA ratio	1.7 ± 0.0^c^	1.8 ± 0.0^b,c^	2.0 ± 0.0^b^	1.9 ± 0.0^b,c^	2.5 ± 0.1^a^	<0.001

1 Values are means ± SEMs, *n* = 8. Totals include some minor components not shown. Labeled means in a row without a common letter differ, *P* < 0.05. C, control; COH, EPA-rich *Camelina* oil, high dose; COL, EPA-rich *Camelina* oil, low dose; FOH, EPA-rich fish oil, high dose; FOL, EPA-rich fish oil, low dose.

2 Derived by using 1-factor ANOVA followed by Tukey’s multiple-comparison tests.

3 Contains n–9 and n–7 isomers.

Muscle EPA concentrations were <0.5%, with no impact of diet on EPA or DHA content (**Table 3**). Total n–3 PUFAs were not different between treatments, and the n–3 to n–6 ratio was higher after the FOH diet (*P* = 0.037) than after the C diet. This difference in the n–3 to n–6 ratio was the result of the low concentration of total n–6 PUFAs observed in the FOH group (*P* = 0.016), attributable to lower concentrations of LA metabolites (20:2n–6, 20:3n–6, and 20:4n–6; *P* < 0.001). No significant impact of treatment on muscle SFA content was observed.

**TABLE 3:** Main FAs (≥0.5 g/100 g FAs) in muscle total lipids of male C57BL/6J mice after 10 wk of feeding diets providing EPA as EPA-rich transgenic *Camelina* oil or EPA-rich fish oil relative to a control diet^1^

	Diet, g/100 g FAs					
FAs	C	COL	COH	FOL	FOH	*P*^2^
14:0	1.1 ± 0.1^b^	1.2 ± 0.0^a,b^	1.4 ± 0.0^a^	1.3 ± 0. 0^a,b^	1.4 ± 0.0^a^	0.010
16:0	22.9 ± 0.2	23.8 ± 0.2	24.3 ± 0.8	23.9 ± 0.7	24.2 ± 0.3	NS
16:1n–7	8.3 ± 0.9	11.3 ± 0.8	10.1 ± 1.1	9.0 ± 1.0	11.0 ± 1.2	NS
18:0	6.4 ± 0.6	5.3 ± 0.4	5.6 ± 0.8	6.6 ± 1.1	4.1 ± 0.5	NS
18:1^3^	41.3 ± 1.2^a^	36.7 ± 1.3^a,b^	30.9 ± 2.0^b^	38.6 ± 2.8^a,b^	41.4 ± 1.7^a^	0.017
18:2n–6	6.6 ± 0.3	7.0 ± 0.3	9.5 ± 0.8	7.0 ± 0.2	7.0 ± 0.4	NS
20:1n–9	0.8 ± 0.1	0.6 ± 0.0	1.3 ± 0.2	0.7 ± 0.0	0.8 ± 0.1	NS
20:2n–6	0.8 ± 0.1^a^	0.4 ± 0.0^b^	0.4 ± 0.0^b^	0.5 ± 0.1^a,b,c^	0.1 ± 0.0^c^	0.002
20:3n–6	0.9 ± 0.1^a^	0.8 ± 0.1^a^	0.7 ± 0.1^a,b^	0.7 ± 0.1^a,b^	0.2 ± 0.0^b^	0.013
20:4n–6	5.4 ± 0.7^a^	4.6 ± 0.4^a^	3.0 ± 0.5^a,b^	4.1 ± 0.7^a^	1.0 ± 0.2^b^	0.002
22:1n–9	0.0 ± 0.0^b^	0.1 ± 0.0^b^	0.6 ± 0.0^a^	0.1 ± 0.0^b^	0.5 ± 0.0^a^	0.000
24:1	0.4 ± 0.0^b^	1.1 ± 0.1^a^	3.0 ± 0.5^a^	0.9 ± 0.2^a,b^	1.6 ± 0.3^a,b^	0.001
22:6n–3	4.0 ± 1.4	6.3 ± 0.8	6.5 ± 1.2	5.7 ± 1.2	6.0 ± 1.0	NS
Total n–3 PUFAs	4.5 ± 0.3	6.6 ± 0.8	8.9 ± 1.1	6.0 ± 1.2	6.4 ± 1.0	NS
Total n–6 PUFAs	14.0 ± 1.0^a^	13.0 ± 0.8^a^	13.7 ± 0.4^a^	12.6 ± 0.8^a,b^	8.4 ± 0.3^b^	0.016
n–3 to n–6 PUFA ratio	0.3 ± 0.0^b^	0.5 ± 0.0^a,b^	0.7 ± 0.1^a,b^	0.5 ± 0.1^a,b^	0.8 ± 0.1^a^	0.037

1 Values are means ± SEMs, *n* = 8. Totals include some minor components not shown. Labeled means in a row without a common letter differ, *P* < 0.05. C, control; COH, EPA-rich *Camelina* oil, high dose; COL, EPA-rich *Camelina* oil, low dose; FOH, EPA-rich fish oil, high dose; FOL, EPA-rich fish oil, low dose.

2 Derived by using 1-factor ANOVA followed by Tukey’s multiple-comparison tests.

3 Contains n–9 and n–7 isomers.

#### Gene expression.

**FIGURE 3:**
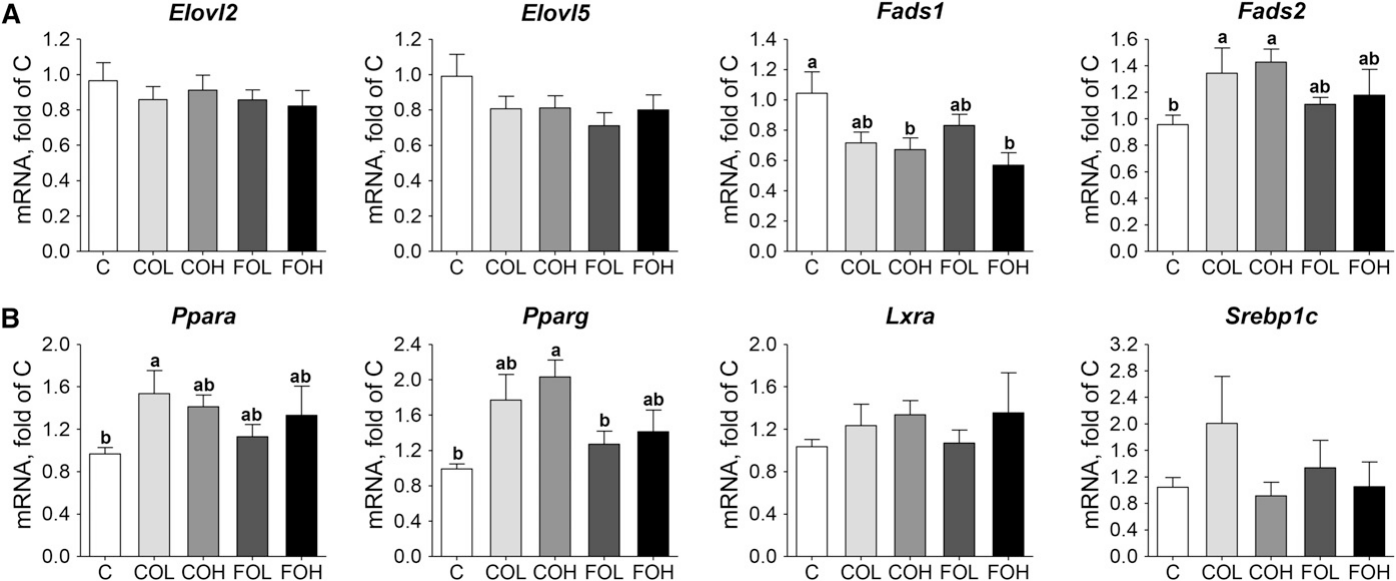
Expression measured by qPCR of long-chain PUFA biosynthesis pathway genes (A) and transcription factor genes (B) in livers of male C57BL/6J mice after 10 wk of feeding diets providing EPA as EPA-rich transgenic *Camelina* oil or EPA-rich fish oil relative to a control diet. Values are normalized expression ratios (means ± SEMs; *n* = 6) relative to the mean expression in mice fed the control diet. Statistical differences were determined by a randomization test (REST). Labeled means without a common letter differ, *P* < 0.05. C, control diet; COH, EPA-rich *Camelina* oil, high dose; COL, EPA-rich *Camelina* oil, low dose; *Elovl2*, elongation of very-long-chain FA 2;* Elovl5*, elongation of very-long-chain FA 5; *Fads1*, FA desaturase 1;* Fads2*, FA desaturase 2; FOH, EPA-rich fish oil, high dose; FOL, EPA-rich fish oil, low dose; *Lxra*, liver X receptor α; *Ppara*, peroxisome proliferator–activated receptor α; *Pparg*, peroxisome proliferator–activated receptor γ; *Srebp1c*, sterol regulatory element-binding protein 1c.

There were no significant differences in the expression of *Elovl2* and *Elovl5* between treatments. Relative to the C group, the expression of *Fads1* was significantly lower in the COH and FOH groups (*P* = 0.015), with COL and COH feeding resulting in higher *Fads2* expression (*P* = 0.045) (**Figure 3A**).

No significant impact of treatment on *Lxra* and *Srebp1c* gene expression was observed (Figure 3B). A significant impact of diet on *Ppara* (*P* = 0.04) and *Pparg* (*P* = 0.013) expression was observed, with higher expression for the COL compared with the C diet for *Ppara* and the COH compared with the C diet for *Pparg*.

## Discussion

Although the impact of EPA and DHA on overall mortality and the incidence and risk of a number of chronic diseases are well recognized (43), the supply of EPA and DHA is limited, and there is a great need to identify alternative and sustainable sources of these beneficial FAs. Given the technological and nutritional limitations associated with alternative sources under investigation (microalgae, zooplankton, SDA-rich oils, etc.), transgenic oilseed crops currently present the most effective solution for the large-scale production of these n–3 LC-PUFAs (14). Here we show for the first time, to our knowledge, that EPA-rich CO (20% total FAs as EPA), when used as a dietary supplement in mice and providing doses physiologically relevant to humans, is a bioavailable source of EPA, resulting in comparable enrichment of EPA in the liver.

The oils were well tolerated, with no deaths during the study, and the mice were in apparent good health. Previous studies in C57BL/6J mice have shown that the inclusion of FO in the diet is accompanied by weight loss (44, 45). However, in the present study, higher EPA-rich CO and FO intakes (COH and FOH), as part of a 10% of energy from fat diet, were associated with body weight gain, and no differences were observed in liver weight. These findings are more consistent with previous studies in which FO was fed to mice (46, 47) and rats (48) as part of low-fat regimes, which showed no major differences in body and liver weight.

In the present study, and independent of oil source, n–3 LC-PUFA supplementation decreased fasting glucose concentrations in a dose-dependent manner. These observations are in line with previous rodent studies (49), but in humans FO supplementation has not consistently affected glucose concentrations (50–53). With regard to serum lipids, feeding mice CO and FO did not modify TC, HDL-cholesterol, or TG concentrations. The TG-lowering effect of FOs in both normolipidemic and hypertriglyceridemic individuals is well documented (54, 55). In contrast and similar to our results, no differences in plasma TGs or TC have been reported previously in mice fed low doses of FO as part of low-fat treatments (56). Significant differences in lipoprotein profiles and metabolism between rodents and humans are likely to explain, in large part, the interspecies differences in responsiveness of serum lipids to EPA and DHA supplementation.

The effect of regiospecificity of dietary TG bioavailability has been previously studied (29, 30), indicating a preferential absorption of FAs when present at the TG *sn*-2 position. Moreover, FO functional characteristics seem to be related to the positional distribution of EPA and DHA on TGs (29, 57). For example, in a recent study in C57BL/6J mice by Yoshinaga et al. (29), DHA in the *sn-*2 position was associated with greater liver DHA accumulation and serum and liver TG lowering, whereas EPA in the *sn*-1 and -3 positions had a greater impact on lowering hepatic cholesterol concentrations. In the present study, regiospecific analysis showed similar proportions of EPA in the *sn*-2 position of TGs in the CO and FO. This is consistent with the uniform response of serum lipids and tissue EPA concentrations reported in mice fed the FO- and CO-based diets.

Our results showed that the EPA-rich CO was as efficient as FO at enriching liver EPA in a dose-dependent manner. Many rodent studies reported increases in hepatic EPA and DHA after FO supplementation, with the enrichment being variable and dependent on the EPA+DHA dose, the EPA to DHA ratio, and the overall composition of the diets (48, 58, 59). Increases in EPA, observed after COH and FOH feeding, were comparable to those reported for the phospholipid fraction of liver from mice fed an FO providing comparable EPA intakes (59).

Previously, dietary EPA, although significantly elevating hepatic and circulating EPA in mice, had only modest effects on DHA accumulation (60). Consistent with these results, the Japan EPA Intervention Study (JELIS) showed that feeding 1.8 g EPA/d had little impact on plasma DHA concentrations in humans (61). In the present study, CO and FO feeding translated into comparable concentrations of liver DHA, with no dose-response evident, showing de novo synthesis in mice that received little (FOL) or no (COL, COH) DHA. The accumulation of DHA in mice fed the EPA-CO can be explained by the high amounts of ALA present in the COL and COH diets, which is characteristic of the CO, and the higher *Fads2* expression observed in the CO groups. This enzyme is responsible for the first and last desaturation steps required to synthesize DHA from ALA (62), and its expression has been shown to be upregulated in the livers of n–3 PUFA–deprived rats (63) and downregulated after feeding n–3 LC-PUFAs (31).

Feeding EPA and DHA in both rodent and human studies resulted in a marked increase in n–3 LC-PUFAs in tissues at the expense of n–6 FAs (64). Similar to previous studies (60), we also observed that dietary DHA, more than EPA, lowered hepatic AA content, which may be partly due to the downregulation of *Fads1* expression observed, which is involved in AA synthesis from LA. Because elongases are also essential in the biosynthesis of LC-PUFAs, we analyzed the effect of CO and FO on the hepatic expression of *Elovl2* and *Elovl5*, and no impact of treatment on their expression was evident. Previously, feeding FO had no effect on *Elovl2* mRNA abundance, but a downregulation on *Elovl5* mRNA levels has been observed (65, 66).

At the transcription level, both desaturases are regulated by the transcription factors *Ppara* and *Srebp1* (67). *Ppara* is a ligand-activated transcriptional factor central to lipid homeostasis and which is influenced by FA status (31, 65, 66). Previous studies have shown that *Ppara* activation is increased by n–3 and n–6 PUFAs (68), and this activation led to increased expression of FA oxidation genes, which resulted in decreased hepatic and plasma TGs (69). In the present study, consistent with these observations, there was a general trend toward increased *Ppara* and *Pparg* after CO and FO feeding, with the etiologic basis of the greatest expression after COL currently unknown.

*Srebp1c* is a major transcriptional factor that regulates enzymes involved in FA and TG synthesis (67). Liver *Srebp1c* expression has been shown to be downregulated in C57BL/6J mice fed FO as part of both high-fat (70) and low-fat (67) regimes. The present results showed no changes in hepatic *Srebp1c* mRNA levels after EPA administration, which was consistent with some previous research in which mRNA expression levels of *Srebp1c* in mice remained unchanged after EPA ethyl ester treatment, although changes in Srebp1c mature protein concentrations were evident (64). *Lxra* represents a further molecular target of dietary PUFAs, which regulates lipogenic gene expression either directly or via regulation of transcription of the *Srebp1c* gene (71). In the present study, no significant differences were found in *Lxra*, which could explain, at least in part, the lack of impact of intervention on *Srebp1c*.

No impact of treatment on brain EPA concentrations was observed, with EPA constituting 0.2% of the FA pool across all groups. This finding is consistent with previous observations, in which, despite comparable potential for transfer across the blood-brain barrier to DHA, EPA represented only a minor component of brain membranes, with supplementation failing to result in EPA enrichment (72). Although some increases in brain DHA were observed, total n–3 PUFAs and the n–3 to n–6 ratio were similar between treatments and less pronounced than in liver.

In contrast to the liver, skeletal muscle EPA concentrations were constitutively low and supplementation with CO and FO had no significant effect on n–3 LC-PUFAs or total n–3 concentrations. Several studies have corroborated that the accumulation of n–3 PUFAs is tissue-dependent (62). The skeletal muscle is mainly involved in lipid oxidation to produce chemical energy. This may explain why DHA is the only n–3 PUFA that accumulates in this tissue given that it has the longest acyl chain length and the highest degree of unsaturation, which can lead to steroisomeric-induced resistance to β-oxidation (73).

In conclusion, EPA from oil derived from genetically modified *Camelina* had comparable effects on liver EPA status, relative to equivalent doses derived from FO. These data could help support any future licensing of this EPA-rich CO for consumption in randomized controlled trials to allow its establishment as a bioavailable and efficacious alternative sustainable source of EPA in humans.

## Footnotes

9 Abbreviations used: AA, arachidonic acid; ALA, α-linolenic acid; C, control; CO, *Camelina* oil; COH, EPA-rich *Camelina* oil, high dose; COL, EPA-rich *Camelina* oil, low dose; *Elovl*, elongation of very long chain fatty acids protein; *Fads*, fatty acid desaturase; FO, fish oil; FOH, EPA-rich Fish oil, high dose; FOL, EPA-rich Fish oil, low dose; HED, human equivalent dose; LA, linoleic acid; LC, long-chain; *Lxra*, liver X receptor α; PO, palm oil; SDA, stearidonic acid; *Srebp1c*, sterol regulatory element-binding protein 1c; TC, total cholesterol; TL, total lipid.
